# A comparative study of wound healing kinetics, efficacy, and profiling of VG111 in canine wounds

**DOI:** 10.3389/fvets.2026.1816520

**Published:** 2026-09-10

**Authors:** Kanwar Arshjot Singh, Paviter Kaur, Savita Attri, Nishant Shekhar, Maninder Karan, Rajneesh Kumar, Pooja Singh, Lipika Singhal, Navdeep Singh, Vikas Gautam

**Affiliations:** 1 Guru Angad Dev Veterinary and Animal Sciences University (GADVASU), Ludhiana, Punjab, India; 2 Postgraduate Institute of Medical Education and Research (PGIMER), Chandigarh, India; 3 University Institute of Pharmaceutical Sciences, Panjab University, Chandigarh, India; 4 Deen Dayal Upadhyaya Gorakhpur University, Gorakhpur, Uttar Pradesh, India; 5 Government Medical College and Hospital Sector-32, Chandigarh, India

**Keywords:** canine wounds, GC–MS, molecular docking, natural compound formulation, povidone-iodine, VG111, wound healing

## Abstract

The management of naturally occurring canine wounds, such as bite and degloving injuries, remains a major veterinary challenge because standard treatments often struggle to balance infection control and tissue regeneration. Although povidone–iodine (PI) remains a widely used first-line antiseptic in both human and veterinary medicine, there are concerns regarding its potential for cytotoxicity and the inhibition of fibroblast growth, which can delay healing. To address these limitations, a prospective comparative study was conducted on 32 client-owned dogs at GADVASU in Ludhiana, where wounds were randomized into four treatment arms. The animals received either the novel natural product formulation VG111 or standard 5% PI, both with and without systemic cefotaxime. Clinical outcomes were assessed through wound contraction percentages and modified Bates-Jensen Wound Assessment Tool (BWAT) scores at 5-day intervals. The results revealed that the VG111-only group achieved complete healing by day 25 nearly twice as fast as the PI group, which took up to 50 days. Interestingly, the addition of systemic cefotaxime to VG111 modestly delayed healing kinetics rather than accelerating them. In addition to clinical evaluation, gas chromatography–mass spectrometry (GC–MS) analysis was utilized to identify 41 chemical constituents within VG111. These chemical findings were further complemented by *in silico* molecular docking simulations, indicating potential mechanisms underlying the formulation’s robust antimicrobial activity.

## Introduction

A wound is described as a disruption in the anatomical, cellular, and functional continuity of the skin or other living tissues (1). Wound healing results from the activity of a complex network of blood cells, cytokines, and growth factors, which leads to the restoration of normal skin and tissue anatomy. To promote wound healing, a wide range of medications, indigenous remedies, and procedures have been employed. These include the topical administration of herbal treatments, natural honey, electromagnetic pulses, fibroblast growth factors, collagen, and topical antibiotics. There is always a need for therapy that can speed up the healing process without interfering with the normal physiological processes involved in wound healing. Additionally, these therapies should provide a safer, more effective, and socioeconomically acceptable treatment with minimum side effects (2).

One of the principal determinants affecting wound healing is the presence of wound infection, which can significantly slow down the healing and often aggravate it. A standard therapeutic treatment strategy involves the application of topical antimicrobials to inhibit the microbial load and facilitate wound healing. However, this very situation highlights the challenges with readily available topical antimicrobials, including adverse effects such as allergic reactions, cytotoxicity, antimicrobial resistance, systemic toxicity, and potential delays in wound healing (3).

These agents include antiseptics such as povidone–iodine (PI), topical antibiotics such as silver sulfadiazine, mupirocin, mafenide acetate, and furacin (4–8). Iodine has been used as an antiseptic to inhibit or kill microorganisms present within a wound or on the intact skin for more than a century, but many concerns have been raised about the use of iodine-based preparations for wound management. There have been concerns regarding allergies, poor penetration, and harmful effects on host cells using PI (9). Despite ongoing developments in wound-care therapy, there is still no ideal solution that can significantly improve wound management, enhance healing, and address the challenges as discussed above (10). Importantly, emerging evidence also suggests that routine topical broad-spectrum antimicrobial application may limit the regenerative aspects of cutaneous repair, reduce wound-induced hair follicle neogenesis (WIHN), and even slow down the overall wound closure process in humans, highlighting the need to balance between antimicrobial control and preservation of pro-regenerative biology of wounds (11).

Natural compound formulations are increasingly explored for wound care because they can combine antimicrobial, antioxidant, and anti-inflammatory properties while offering improved tolerability. VG111 is a patented formulation developed at PGIMER, Chandigarh (Indian patent no. 353978), which has been reported to accelerate tissue repair and demonstrate broad anti-pathogenic activity in humans and companion animals (12, 13). In this prospective comparative study, we evaluated topical VG111 against 5% povidone–iodine in client-owned dogs presenting with naturally occurring wounds. A total of 32 clinical cases were randomly assigned to 4 treatment arms (VG111 ± systemic cefotaxime; PI ± systemic cefotaxime) and followed at 5-day intervals until closure, with wound contraction, modified BWAT scoring, and exudation recorded alongside wound culture at day 0 and day 15. To connect clinical performance with plausible mechanistic drivers, VG111 was chemically profiled by GC–MS and key constituents were assessed by molecular docking against bacterial targets linked to replication and virulence (GyrA, SrtA, and LasR).

## Materials and methods

The study involved 32 client-owned dogs, regardless of sex, breed, and age, all presented with different types of wounds such as bite wounds, traumatic lacerated wounds, ulcerated abscess wounds, suture dehiscence wounds, and degloving wounds. This research was conducted at the Multispecialty Veterinary Hospital, Department of Veterinary Surgery and Radiology, Guru Angad Dev Veterinary and Animal Sciences University, Ludhiana, Punjab. Initially, the total number of dogs was 84 in the study, and 52 dogs were excluded because 5 died during the study period and the owners of 47 dogs did not provide follow-up on their wounds. The study was conducted on 32 dogs in total. The initial cohort of 52 excluded dogs was a non-randomized convenience sample based on clinical presentation, but the final 32 cases were strictly randomized into four treatment arms. Baseline characteristics (including wound type, location, initial severity, etc., on day 0 under the BWAT score) were assessed, and the dogs were randomized to different groups. In addition, the clinical assessors of the macroscopic parameters of healing were blinded to the treatment group.

The dogs were randomly allocated to four treatment groups (Table 1: Grouping of Animals), and a different treatment protocol was followed in each group. Wound treatment was standardized in all groups. At each visit, wounds were irrigated with sterile normal saline to clear debris. The allocated topical treatment (VG111 or 5% v/v PI) was subsequently applied once daily in a standardized volume of 1–2 mL per 5 cm^2^ to completely cover the wound bed. VG111 was kept in airtight containers at room temperature (20–25 °C). Group 1 dogs were treated with topical application of VG111 once in a day till complete wound healing and antibiotic cefotaxime @ 20 mg/kg bd for 5 days, group 2 was treated with VG111 alone, group 3 was treated with topical PI (5% v/v) once in a day till complete wound healing and antibiotic cefotaxime administered intramuscularly (IM) @ 20 mg/kg bd for 5 days and group 4 was treated with topical PI (5% v/v) till complete wound healing. The following parameters were used to compare the four treatment groups.

1 *Wound contraction*: The wound was observed for peripheral wound contraction after treatment. The progressive decrease in wound area was recorded on the 5th, 10th, 15th, 20th days, etc., at 5-day intervals till complete healing. Complete healing was defined as strict complete epithelial continuity, functional recovery of the local tissue, and absence of any exudate.

**Table 1:** Treatment groups and protocols for canine wound management in the study.

Group	No. of animals	Treatment protocol for wounds
Group 1	8	Treated with VG111 + inj cefotaxime
Group 2	8	Treated with VG111 solution
Group 3	8	Treated with povidone–iodine 5% solution + inj cefotaxime
Group 4	8	Treated with povidone–iodine 5% solution

Percentage of wound contraction = $\frac{a-b}{a}\;\;x\;100$

Where, a = Wound area on day zero and b = Wound area on the nth day

The wound area was calculated with ImageJ software.

2 *BWAT scoring*: A macroscopic evaluation of the wound was then assessed every 5 days, taking into account the width, depth, type of necrotic tissue, color of the skin around the wound, borders, type and amount of exudate, edema of the surrounding tissues, and degree of epithelialization. For each characteristic, a 1 to 5 grade was given to each lesion to show its severity. The Bates–Jensen Wound Assessment Tool (BWAT) was modified (14). The healing process was monitored using such a score (wound healing at BWAT score 10). A final number depicting the overall severity of the skin was calculated at every time interval by averaging all results. The overall BWAT value shows real-time information on the wound’s healing status.
3 *Wound exudation*: Exudation at the surgical site was evaluated on the 0th, 5th, 10th, 15th, and 20th day and so on at 5-day interval by gentle touch and arbitrary scores was given as Nil (−), Mild (+) moderate (++) and marked (+++) and the arbitrary score was converted in numeric score as 0, 1, 2, and 3.
4 *Wound microbiology*: At the time of the presentation of the case, a wound swab was taken with a sterile swab for microbial culture. The wound was flushed with sterile normal Saline solution to remove any kind of debris from the wound surface, and a swab was taken under aseptic conditions from the depth of the wound, as deep as possible. The wound microbiology for bacterial identification, culture, and sensitivity testing was done at 0 day (pretreatment) and 15th day of treatment (posttreatment). The antimicrobial susceptibility test (AST) testing was done according to CLSI guidelines with 27 antibiotics.
5 *GC–MS evaluation of VG111 constituents*: VG111, originally formulated as an oil (in a sesame oil vehicle), was analyzed by gas chromatography–mass spectrometry (GC–MS) to determine its chemical composition. The active constituents from the oil matrix, a representative aliquot of the entire VG111 formulation, were extracted using methanol. The methanol extract was then filtered through a 0.22 μm membrane to remove particulate matter. The resulting solution was injected (in split mode) into a GC–MS system equipped with a capillary column (e.g., 30 m × 0.25 mm ID, 0.25 μm film thickness). The oven temperature was programmed to start at 50 °C (held for 2 min) and then increased at a rate of 10 °C/min to a final temperature of 300°C, which was maintained for 10 min. The mass spectrometer was operated in electron ionization (EI) mode with a scan range of 50–550 m/z. Compound identification was performed by matching the acquired spectra against standard mass spectral libraries.
6 *Molecular docking for ligand-binding estimation*: The computational workflow was performed using Schrödinger Drug Discovery Suite to estimate the multi-target inhibitory potential of the formulation against key bacterial survival pathways. The target proteins were GyrA (PDB: 5BS3, resolution: 2.65 Å), SrtA (PDB: 6R1V), and LasR (PDB: 6D6D, resolution: 1.7 Å) (15–17). The receptor grid was generated using the active sites of the respective targets. Ligands were prepared via LigPrep with the OPLS3e force field at a physiological pH of 7.0 ± 2.0. Target proteins were refined using the Protein Preparation Wizard to optimize hydrogen-bond networks and correct structural artifacts. Virtual screening was conducted with Glide XP, followed by Prime MM–GBSA rescoring to estimate binding free energies ΔG*_bind_* using the VSGB 2.0 solvation model (18, 19).
7 *Statistical analyses*: Wound contraction percentages were calculated to normalize raw wound areas to day 0 baseline values, standardizing different initial wound sizes for accurate proportional comparison. Clinical assessments (BWAT and exudation scores) for each randomized cohort (*n* = 8) were pooled and presented as Mean ± Standard Error (SE). The summarised data were analyzed statistically using R (v4.3.1). Baseline comparability (Day 0) and subsequent pairwise group comparisons across time intervals were performed via independent two-sided Student’s *t*-tests, based on the group standard errors. Exact test statistics and *p*-values are reported throughout the results, with statistical significance accepted at *p* < 0.05.

## Results

### Percent wound contraction

**Figure 1:**
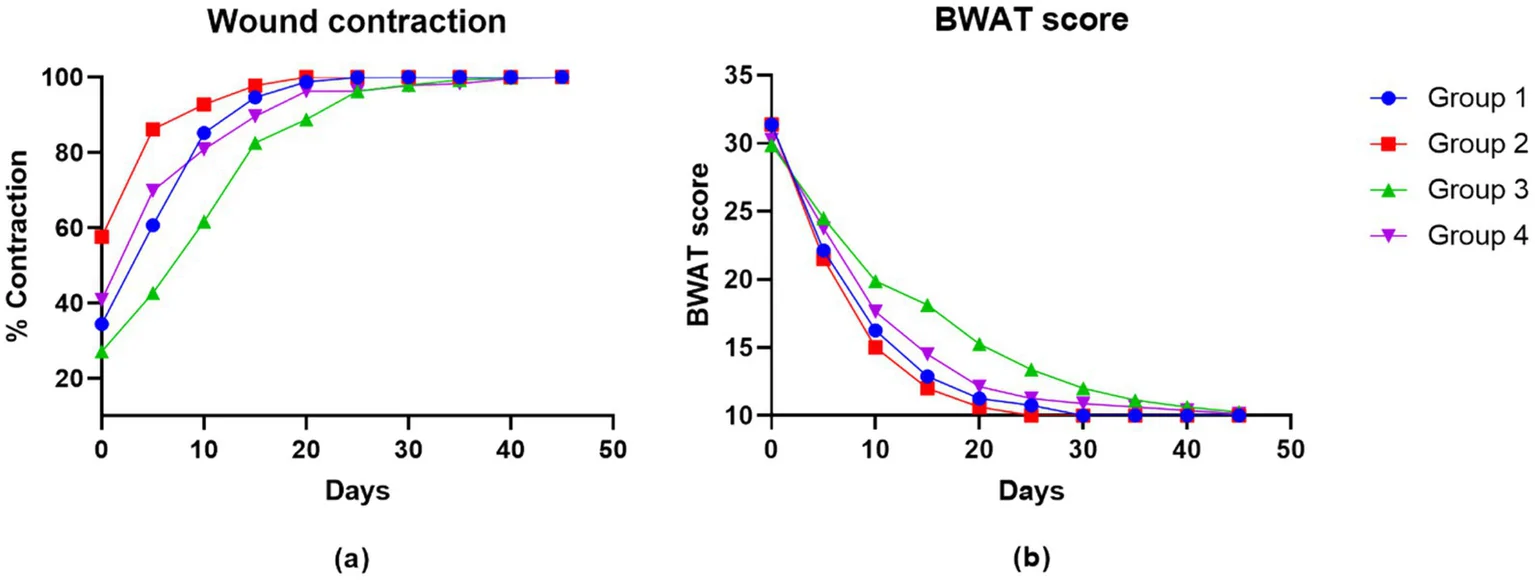
**(a)** Wound contraction and **(b)** BWAT score of different treatment groups as described in Table 1 upon application for 50 days.

A measure of healing progress is the percentage of wound contraction. It is a criterion that is not affected by the extent, depth, or edema of the wound. Wound contraction was measured at 5-day intervals (5, 10, 15, 20, and so on) until the wound was completely healed. The four study groups showed significant differences in the rate of wound healing. The maximum rate of wound contraction was observed in group 2 (VG111 alone) on day 5, which was 57.60 ± 0.12%, significantly higher than that observed with the standard treatment, PVPI, in group 4 (40.82 ± 0.08%; *t* = 116.0, df = 14, *p* < 0.0001). Moreover, independent pairwise analysis confirmed that systemic cefotaxime significantly delayed the early wound contraction. Group 2 showed faster healing than group 1 (34.42 ± 0.11%; *t* = 142.0, df = 14, *p* < 0.0001), and similarly, group 4 showed faster healing than group 3 (27.12 ± 0.17%; *t* = 72.9, df = 14, *p* < 0.0001). On the contrary, group 2 achieved 100% wound contraction within 25 days, whereas groups 3 and 4 achieved 100% contraction within 50 days (Figure 1a and Table 2).

**Table 2:** Average wound contraction percentage (mean ± SE) for each group at each time interval.

Days	Rate of wound contraction			
	Group1 (VG111 + cefotaxime)	Group2 (VG111)	Group3 (5% PI + cefotaxime)	Group4 (5% PI)
D5	34.42 ± 0.11	57.60 ± 0.12	27.12 ± 0.17	40.82 ± 0.08
D10	60.63 ± 0.12	86.15 ± 0.23	42.63 ± 0.12	69.82 ± 0.09
D15	85.20 ± 0.20	92.78 ± 0.32	61.66 ± 0.13	80.83 ± 0.12
D20	94.62 ± 0.34	97.75 ± 0.96	82.55 ± 0.15	89.63 ± 0.15
D25	98.76 ± 0.80	100 ± 00	88.81 ± 0.19	96.27 ± 0.26
D30	99.95 ± 0.95	100	96.30 ± 0.43	96.32 ± 0.27
D35	100	100	97.97 ± 0.73	97.82 ± 0.45
D40	100	100	99.27 ± 2.06	98.33 ± 0.60
D45	100	100	99.88 ± 8.88	99.68 ± 3.2
D50	100	100	100	100

Exact *p*-values for key pairwise comparisons across groups were calculated using independent two-sample *t*-tests (*p* < 0.05 considered significant).

### Average BWAT score

Many factors influence the overall severity of a wound, and the BWAT approach has been applied to assess these factors. The BWAT approach allowed a comprehensive evaluation of the lesions for each parameter studied by pooling the scores associated with each criterion at each time point. The mean score at day 0 was 31.38 for group 1, 31.38 for group 2, 29.38 for group 3, and 30.25 for group 4. At the beginning of treatment, BWAT scores did not differ significantly among all the groups, confirming that baseline wound severity was well balanced between the randomized cohorts (group 2 vs. group 4: *t* = 0.669, df = 14, *p* = 0.514; group 1 vs. group 3: *t* = 1.30, df = 14, *p* = 0.214). After 15 days of treatment, the BWAT score was significantly reduced from day 0 to day 15 in all four groups. BWAT for group 2 was improved by 21.37 in 25 days of treatment and became 10 (shows total wound healing), followed by group 1 at 10.75, group 4 at 11.25, and group 3 at 13.37 on the 25th day, showing slower healing. Groups 3 and 4 required 50 days to reach the 10 BWAT score. The BWAT score at day 0 was statistically similar, and after the end of treatment, the score was significantly different between groups 1, 2, 3, and 4 (Figure 1b and Table 3).

**Table 3:** Average BWAT score (mean ± SE) at different time intervals in four groups.

Days	BWAT scores			
	Group1 (VG111 + cefotaxime)	Group2 (VG111)	Group3 (5% PI + cefotaxime)	Group4 (5% PI)
D0	31.38 ± 0.78	31.38 ± 1.04	29.88 ± 0.85	30.25 ± 1.33
D5	22.13 ± 0.63	21.50 ± 0.97	24.50 ± 0.83	23.75 ± 0.60
D10	16.25 ± 0.64	15.00 ± 0.71	19.88 ± 0.56	17.63 ± 0.46
D15	12.88 ± 0.78	12.00 ± 1.18	18.13 ± 0.46	14.50 ± 0.53
D20	11.25 ± 0.90	10.63 ± 1.60	15.25 ± 0.44	12.13 ± 0.58
D25	10.75 ± 1.33	10.00	13.38 ± 0.54	11.25 ± 0.80
D30	10.00	10.00	12.00 ± 0.81	10.88 ± 1.14
D35	10.00	10.00	11.13 ± 1.14	10.63 ± 1.60
D40	10.00	10.00	10.63 ± 1.60	10.38 ± 2.67
D45	10.00	10.00	10.25 ± 4.0	10.13 ± 8.0
D50	10.00	10.00	10.00	10.00

### Wound exudation

Wound exudation ranged from mild to moderate in most cases across all four groups. On day 0, the mean exudation scores were 1.12 in group 1, 1.87 in group 2, 1.50 in group 3, and 1.25 in group 4. Exudation resolved completely by day 15 in group 2 and by day 25 in group 3, whereas groups 1 and 4 required more than 30 days to reach a nil exudate level (Table 4). Between-group comparisons did not show a statistically significant difference in exudation scores at the assessed time points. In contrast, within each group, mean exudation scores declined significantly over time, with the steepest reduction observed up to day 20. Although the between-group differences were not significant, group 2 showed the earliest clinical resolution of exudation.

**Table 4:** Level of wound exudation (mean ± SE) at different time intervals in all treatment groups.

Days	Exudation				Mean ± SE
	Group1 (VG111 + cefotaxime)	Group2 (VG111)	Group3 (5% PI + cefotaxime)	Group4 (5% PI)	
D0	1.12	1.87	1.50	1.25	1.43
D5	1.00	0.87	1.25	0.87	0.96
D10	0.25	0.25	0.75	0.37	0.40
D15	0.25	0	0.37	0.37	0.25
D20	0.12	0	0	0.12	0.06
D25	0	0	0	0.12	0.06
Total	0.48	0.50	0.62	0.52	0.53

## Microbiological studies of wounds

### Bacterial isolation and identification

#### Pretreatment sample

A total of 32 pretreatment wound swab samples were processed for the isolation of bacteria, of which 26 (81.25%) yielded positive bacterial culture, whereas 6 (18.75%) samples showed no bacterial growth (Table 5). Of the 26 isolates identified in this study, 21 (80.7%) were Gram-positive, and 5 (19.3%) were Gram-negative. The most frequently isolated bacterial genus was *Staphylococcus* (16/26; 61.5%). To further characterize the clinical infection profile, the isolates were classified according to their clinical significance as recognized opportunistic pathogens (14/26; 53.8%) and resident skin commensals or low-virulence organisms (12/26; 46.2%). The most commonly identified pathogens were highly virulent coagulase-positive Staphylococci (*Staphylococcus pseudintermedius* [7.7%], *Staphylococcus intermedius* [7.7%], *Staphylococcus aureus* [7.7%], and *Staphylococcus schleiferi* [3.8%]) and opportunistic Gram-negative and enteric isolates, including *Pseudomonas aeruginosa* [7.7%], *Escherichia coli* [7.7%], *Enterococcus faecalis* [7.7%], and *Proteus* spp. The rest of the skin commensals, including coagulase-negative *Staphylococcus* (CoNS), were *Staphylococcus simulans* (15.4%), *Staphylococcus warneri* (11.53%), *Staphylococcus haemolyticus* (7.7%), *Macrococcus canis* (7.7%), and *Micrococcus luteus* (3.8%).

**Table 5:** Group-wise pretreatment microbiology of all four groups.

Group1 (VG111 + cefotaxime)		Group2 (VG111)		Group3 (5% PI + cefotaxime)		Group4 (5% PI)	
C11	*Staphylococcus simulans*	C21	*Macrococcus canis*	C31	*Staphylococcus pseudointermedius*	C41	*Staphylococcus simulans*
C12	*Staphylococcus simulans*	C22	*Staphylococcus aureus*	C32	*no growth*	C42	*Macrococcus canis*
C13	No growth	C23	*Escherichia coli*	C33	no growth	C43	*Staphylococcus haemolyticus*
C14	*Pseudomonas aeruginosa*	C24	*Staphylococcus aureus*	C34	*Micrococcus luteus*	C44	*Staphylococcus pseudintermedius*
C15	*Staphylococcus schleiferi*	C25	*Staphylococcus simulans*	C35	*Staphylococcus haemolytic*	C45	*Escherichia coli*
C16	*no growth*	C26	*Enterococcus faecalis*	C36	*Proteus*	C46	No growth
C17	*Pseudomonas aeruginosa*	C27	*Staphylococcus intermedius*	C37	*Staphylococcus warnerii*	C47	*Staphylococcus warnerii*
C18	*Staphylococcus warnerii*	C28	*Enterococcus faecalis*	C38	*Staphylococcus intermedius*	C48	No growth

#### Microbiology after treatment

On day 15, 13 posttreatment samples were analyzed across the four treatment groups. Cultures were positive only in three samples (23.0%), all of which were Gram-positive. One case (group 4) showed persistent clinical signs of infection, with *Staphylococcus pseudintermedius* isolated at both pre and posttreatment time points. The other two culture-positive cases showed no clinical signs of infection. In both cases, the microbial profiles differed from those of the respective baseline cultures but consisted of organisms considered part of the normal commensal flora. In one case (group 1), the culture changed from negative at baseline to *Staphylococcus epidermidis*, whereas in the other case (group 2), the culture changed from *Staphylococcus simulans* to *Staphylococcus epidermidis*.

#### Culture sensitivity test for the isolates

All 14 positive isolates of *Staphylococcus* spp., 2 isolates of *Pseudomonas aeruginosa*, and 2 isolates of *E.coli* were subjected to culture and antimicrobial susceptibility testing, and results were interpreted according to CLSI guidelines (2021) (20).

**Figure 2:**
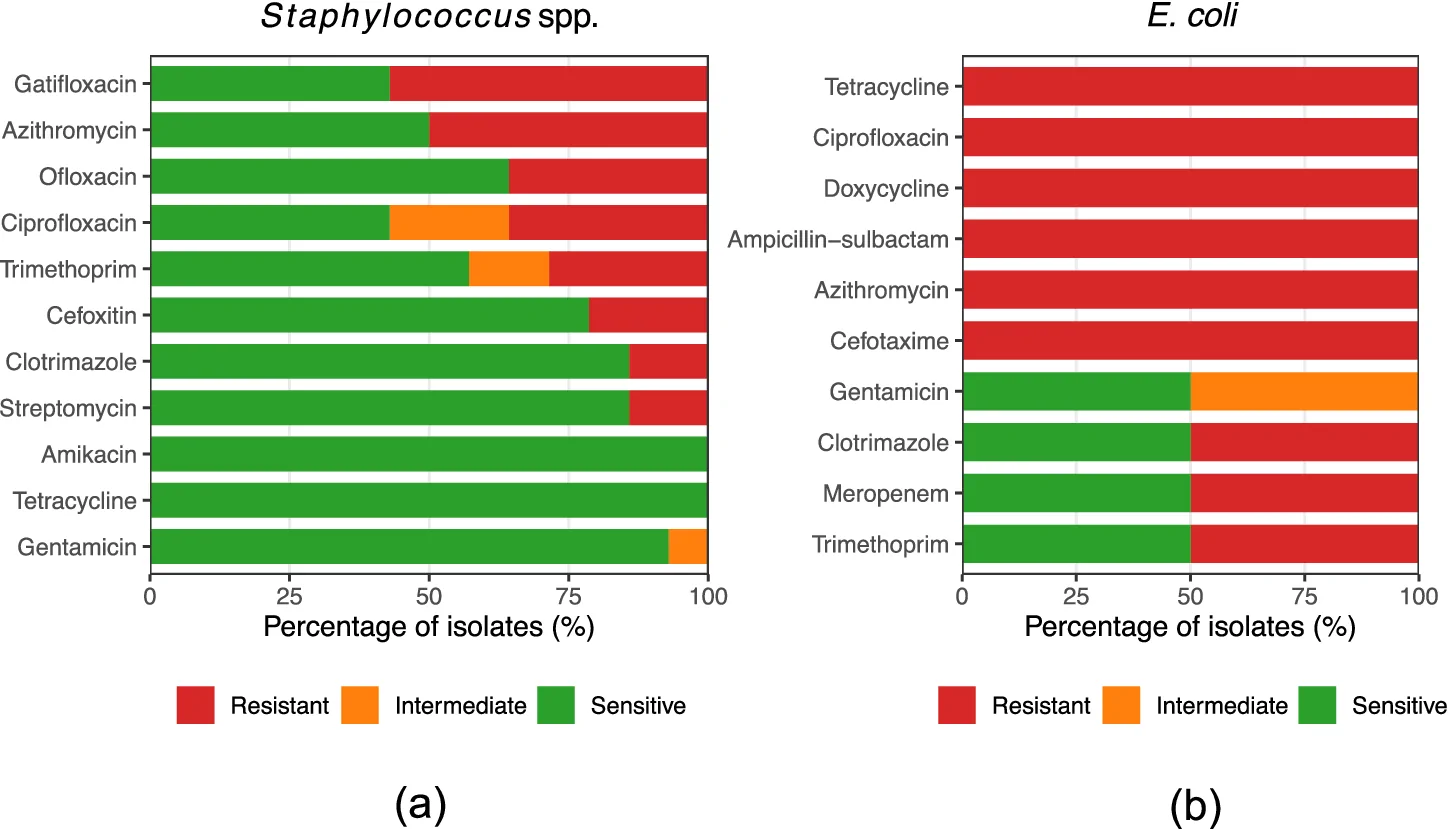
**(a)** Antibiotic sensitivity profile of all *Staphylococci* isolated from the canine wounds; **(b)** Antibiotic susceptibility profile of *E. coli* isolated from canine wounds.

The antibiogram study revealed that *Staphylococcus* spp. showed the highest resistance to gatifloxacin (57.1%), followed by azithromycin (50%), ciprofloxacin and ofloxacin (35.7%), and trimethoprim (28.5%), cefoxitin (21.4%), streptomycin and clotrimazole (14.2%). The *Staphylococcus* spp. were 100% susceptible to tetracycline and amikacin, followed by 92.9% sensitive to gentamicin (Figure 2a).

*E. coli* was tested for resistance or susceptibility against 13 antibiotics. All the *E. coli* isolates were 100% resistant to cefotaxime, azithromycin, ampicillin–sulbactam, doxycycline, ciprofloxacin, tetracycline, and 50% were susceptible to trimethoprim, meropenem, and clotrimazole (Figure 2b: AST of *E. coli* isolates) Intermediate resistance (50%) was seen against gentamicin.

#### GC–MS analysis

**Figure 3:**
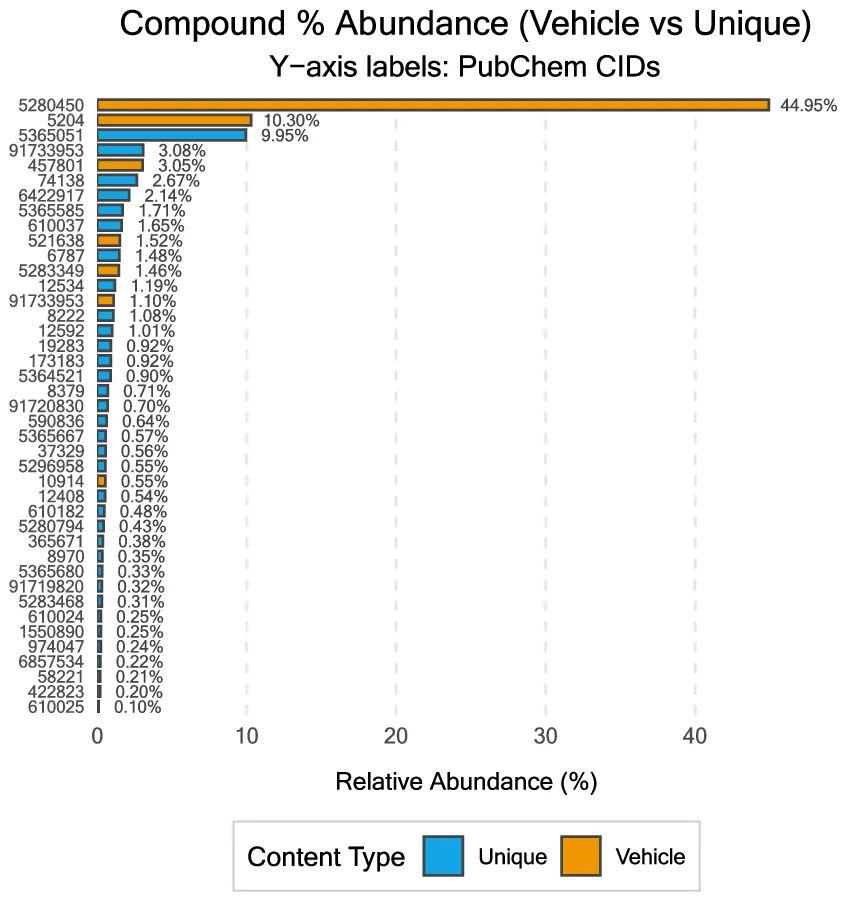
Horizontal bar plot showing the relative abundance (%) of chemical constituents identified in VG111 by gas chromatography–mass spectrometry. Orange bars indicate constituents associated with the sesame oil (vehicle), whereas blue bars indicate constituents unique to the herbal compounds of the formulation.

The GC–MS analysis of VG111 revealed a total of 41 significant chemical constituents, representing 96.99% of the detected composition (Figure 3). The major compounds identified were 9,12-Octadecadienoic acid (Z, Z)-(or linoleic acid) (44.95%), 2,6-Bis(3,4-methylenedioxyphenyl)-3,7-dioxabicyclo(3.3.0)octane (or asarinin) (10.30%), 3-eicosene, (E) (9.95%), and *γ*-sitosterol (3.05%). Together, these major constituents accounted for approximately 68.25% of the total composition, whereas compounds such as ar-turmerone (0.21%), stigmasterol (0.43%), campesterol (0.92%), and cholestane (0.22%) were present in minor amounts.

#### *In silico* protein-ligand binding analysis

**Figure 4:**
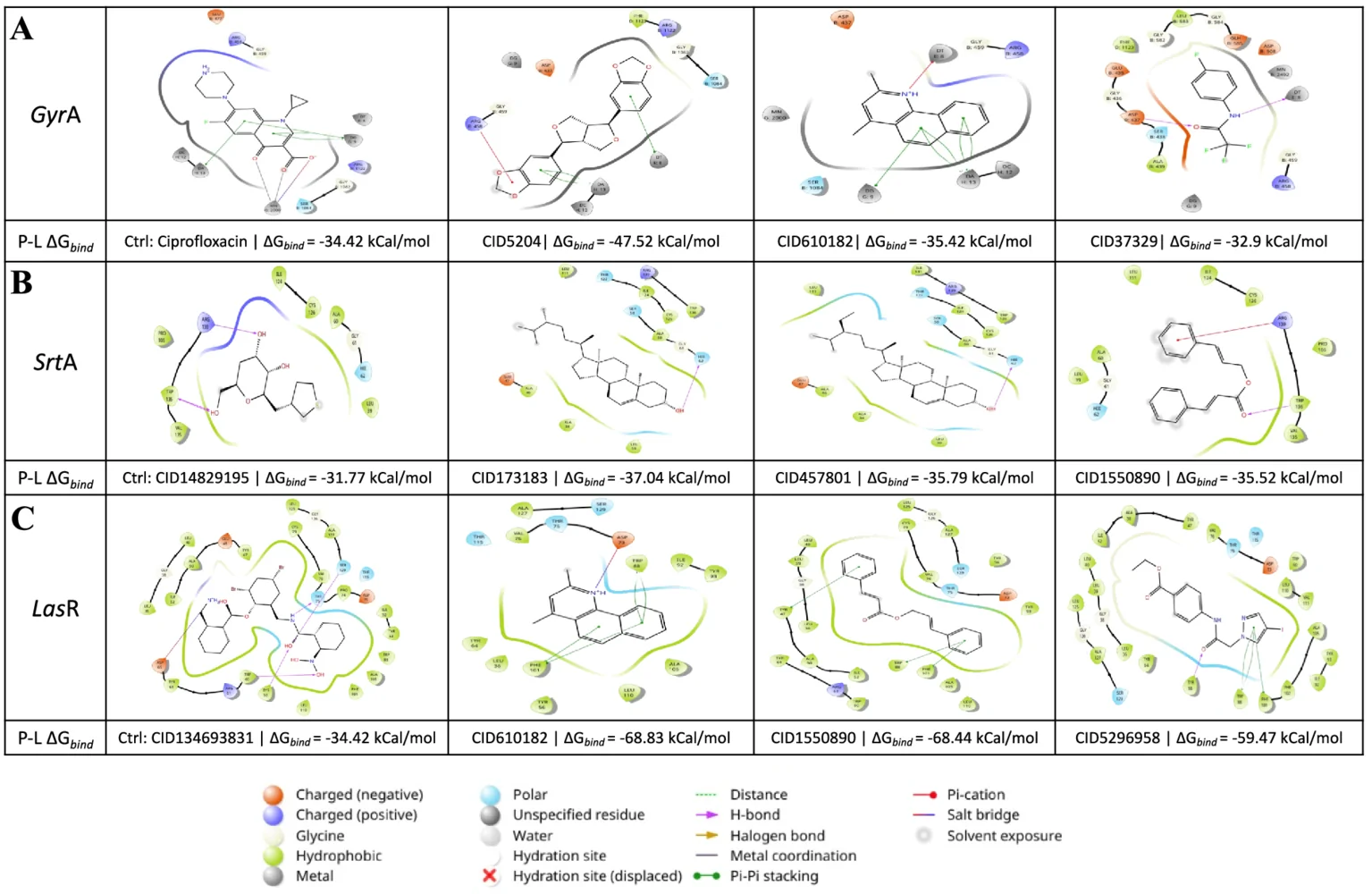
Molecular docking and MM-GBSA analysis of selected VG111 constituents against bacterial targets. **(A)** Predicted interactions with DNA gyrase subunit A (GyrA), **(B)** interactions with *Staphylococcus aureus* sortase A (SrtA), and **(C)** interactions with the *Pseudomonas aeruginosa* quorum-sensing regulator LasR. Representative ligand–protein binding poses, interaction profiles, docking scores, and MM-GBSA binding free-energy estimates are shown for selected VG111 constituents alongside the respective reference compounds.

MM-GBSA analysis suggested several leads exhibit highly favorable estimated binding free energies compared with standard clinical controls (Figure 4). GyrA Target: Sesamin (CID5204) achieved an estimated ΔG*_bind_* of −47.52 kcal/mol, a predicted +38.0% affinity increase over ciprofloxacin (−34.42 kcal/mol). SrtA Target: Phytosterols Campesterol (CID173183) and γ-Sitosterol (CID457801) yielded predicted energies of −37.04 kcal/mol and −35.79 kcal/mol, respectively, outperforming the control (−31.77 kcal/mol). Finally, LasR Target: Benzo[h]quinoline (CID610182) and Cinnamyl cinnamate (CID1550890) were identified as “super-binders” with scores near −69 kcal/mol, effectively doubling the affinity estimated for the control (−34.42 kcal/mol).

## Discussion

The primary objective of this study was to evaluate the clinical efficacy, wound healing kinetics, and antimicrobial activity of VG111, a novel natural product formulation, in dogs with wounds presented for clinical treatment. For performance benchmarking, standard 5% povidone–iodine (PI) was used as a comparator, as it continues to be a widely used topical agent globally because of its availability and broad-spectrum antiseptic activity. We report changes in the wound physiology of 32 dog wounds in terms of wound size contraction, exudation, and BWAT score, which Drudi et al. (21) described as an excellent method for the objective assessment of healing across wounds with varying clinical presentations. It was observed that percentage wound contraction and BWAT score followed similar trends, indicating significant healing in the VG111 arm, i.e., groups 1 and 2, compared with the PI arm, i.e., groups 3 and 4. The percentage of wound contraction showed complete closure of wounds between 25 and 30 days in the VG111 arm compared with the PI arms, i.e., 45–50 days. The change in BWAT score also showed a similar trend, and within 25 days the VG111 arm reached a BWAT score of 10, while groups 3 and 4 took 45–50 days to reach the same score, making PI 20–25 days slower than the VG111. This result was further supported by a study by Balin and Pratt (22), which demonstrated inhibition of skin fibroblast growth by PI at 0.1 and 1.0% dilutions. Similar results were reported for PI (5%) solution by Das and Nagar et al. (23, 24). Deore et al. and Singh et al. (25, 26) also reported similar wound healing percentages in calves treated with PI. Gautam et al. (27) described povidone–iodine as comparatively less effective than propolis for infected wound healing in dogs. Noboga and Davane (28) described how various herbal formulations accelerate wound healing by enhancing epithelialization, neovascularization, collagen synthesis, wound contraction, granulation tissue formation, and tensile strength.

Though not found to be significant, the rate of exudate reduction was higher with VG111 than in the PI groups; nonetheless, exudates were controlled within 25 days in all four groups. This “earlier drying” in the VG111 arms is clinically relevant because persistent exudation is often what keeps a wound in a chronic inflammatory state. In a study by Sharma et al. (2), the authors found that the mixture of herbs was responsible for the faster reduction of exudate than PI and herbs used alone in dog cutaneous wounds, and this difference was statistically significant (Supplementary Section S1 wound images; Supplementary Figures SF1–SF4). A similar finding was observed by Kavitha et al. (29), who stated that the anti-inflammatory and antibacterial activities of polyherbal medication resulted in reduced exudation from the wound.

This study also investigated the effect of systemic antibiotics, i.e., cefotaxime, on the rate of wound healing. Among the four groups of canine cases defined above, it should be noted that group 2, i.e., the VG111 alone, has the highest rate of wound contraction and achieves 100% contraction in 25 days, while the average contraction period for group 1, i.e., VG111 + cefotaxime inj. was between 31 and 35 days (Supplementary Section S1 wound images; Supplementary Figures SF1, SF2). Furthermore, there was a significant 5-day difference between the two groups in the time taken to reach a BWAT score of 10, suggesting that the use of cefotaxime caused a slight delay (30). Our findings also suggest that antibiotic intervention may delay wound healing, consistent with previous reports (30). This delay is likely mediated, at least in part, by disruption of the local commensal skin microbiome. Commensal bacteria play an important role in inducing pro-regenerative immune responses and tissue neogenesis, and systemic broad-spectrum antibiotics may inadvertently suppress these physiological healing triggers (11). However, this relationship appears to be more complex, as Khadka et al. (31) demonstrated that both antibiotic-mediated microbiome depletion and excessive topical exposure to commensal bacteria delayed skin barrier repair, highlighting that a balanced skin microbiome, rather than simple bacterial presence or absence, is essential for optimal healing. Advanced adjunctive treatments such as negative pressure wound therapy, beta-blockers, and laser therapies are increasingly being incorporated into modern wound care to overcome such delays in epithelialization (32, 33). Similarly, within the PI-treated groups, Group 3 (PI + cefotaxime) showed a slower wound-healing rate than Group 4 (PI alone), though the difference in wound closure between the two groups was not statistically significant. But interestingly, it was observed that PI + cefotaxime showed delayed control of exudation, substantiating the delay of wound healing (Supplementary Section S1 wound images; Supplementary Figures SF1–SF4).

The bacterial isolation findings of the study were not entirely consistent with those reported by Urumova et al. and Padhy et al. (34, 35). In both studies, the most prevalent bacterium isolated from traumatic and postoperative wounds was *S. intermedius*, whereas the present study showed *S. simulans* as the most prevalent bacterial isolate and *S. intermedius* as the 3rd most common bacterial isolate. Hence, the reported prevalence cannot be considered a true estimate of prevalence. However, the results were consistent with the present study, which showed *Staphylococcus* as the most prevalent genus found in all kinds of wounds. The prevalence of other bacterial isolates obtained was nearly similar to that of the present study. Griffin et al. (36) reported similar kinds of observations regarding the isolation of *E. coli* (13%), *P. aeruginosa* (5%), and *Proteus* (5%). In the present study, the technique used for bacterial identification was MALDI–TOF, which enabled identification of all bacterial species that can be missed in routine biochemical identification and other conventional tests. Posttreatment in groups 1, 2, and 3, no positive culture associated with wound infection was found, but in group 4 (5% PI), one positive case with infection was found after 15 days of treatment. The comparison of pretreatment and posttreatment samples showed that groups 1, 2, and 3 were effective against all bacterial isolates, whereas group 4 was less effective. Nolff et al. (37) described in their study that from the time of presentation to the course of treatment, the number of positive bacterial cultures decreased during the treatment period, as has been observed in this study.

In addition to the wound-healing parameters and antimicrobial efficacy demonstrated in the clinical study, the GC–MS analysis of VG111 provides valuable mechanistic insight into its activity. The chemical profiling revealed a complex blend of bioactive constituents, with linoleic acid (PubChem CID5280450) (44.95%), asarinin (CID5204) (10.3%), 3-eicosene, (E) (CID5365051) (9.95%), and *γ*-sitosterol (CID457801) (3.05%) emerging as the major compounds. Linoleic acid, well known for its role in maintaining membrane fluidity and enhancing skin barrier function (38, 39), is also documented to exhibit potent antibacterial activity against pathogens such as *S. aureus* and various *Bacillus* species (40). This may have contributed to the rapid wound contraction and improved BWAT scores observed in the VG111-treated groups. Similarly, asarinin, a lignan with recognized anti-inflammatory properties, may synergize with γ-sitosterol and stigmasterol, which are known to modulate inflammation and promote tissue repair (41). Alongside these major constituents, a diverse array of minor compounds was detected, including terpenoids, steroids, alcohols, hydrocarbons, organic acids, and esters that may collectively contribute to activity at the wound surface. For example, 1,2-Benzisothiazol-3-amine TBDMS (CID91733953) can contribute to broad-spectrum antimicrobial properties, and tetracosane has antibacterial activity and structural stability (42). 2,4-Decadienal (E, E) (CID5283349) has shown antibacterial effects against *S. aureus*, *E. coli*, and *P. aeruginosa*, and hexadecenoic acid exhibits antifungal activity against *Candida albicans* and other fungal pathogens (43, 44). In addition, cyclotrisiloxane (CID23657874) (0.55%) has been associated with inhibition of protumorigenic inflammatory and oxidative stress genes. Furthermore, some compounds such as Sesamin (CID72307) and Ar-tumerone (CID558221) are reported to have antiproliferative activity (45). The collective presence of these compounds, effectively extracted from the sesame oil vehicle via methanol extraction, appears to establish a dual modality: direct antibacterial action reducing the microbial burden at the wound site and the promotion of a regenerative environment that accelerates healing. These findings correlate well with our study’s observations, wherein VG111 not only achieved significantly faster wound closure but also demonstrated superior antimicrobial control compared to PI-based treatment.

Importantly, the primary antibacterial action proposed for VG111 is an oxidative effect at the wound interface, overwhelming bacterial cells with ROS and reducing their viability. That ROS-driven mechanism helps explain why the formulation remains effective even when the culture spectrum is mixed and when wounds differ in depth and chronicity. At the same time, we wanted to go one step further: if ROS is the hammer, are there also “locks” that specific constituents might be picking molecular targets that could explain the particularly clean control of exudation and the resistance to chronicity that we saw clinically? This is where the in silico work becomes useful: not as proof of inhibition, but as a way to map plausible secondary targets and generate testable hypotheses. To further elucidate the molecular mechanisms driving this superior antimicrobial control, our in silico docking analysis therefore serves as a hypothesis-generating extension of the formulation’s activity profile. While the primary action involves oxidative stress, computational results suggest certain components could be used as a targeted secondary strike against bacterial survival (12). However, it should be clearly stated that the activities of these individual compounds and their specific docking and MM–GBSA interactions with GyrA, SrtA, and LasR have not been validated *in vitro* or *in vivo* in the present study. We have also observed and reported that VG111 exhibits anti-biofilm activity even against intact/established biofilm architecture, which strengthens the biological plausibility of targeting persistence pathways (and helps connect the computational predictions to the “less exudation, less chronicity” clinical phenotype) (12).

Within this framework, the docking results provide further mechanistic insight (Figure 4). First, a sesamin/asarinin-type lignan consistent with our GC–MS profile demonstrated the strongest predicted interaction with DNA gyrase subunit A (GyrA; Figure 4A), with an estimated ΔGbind of −47.52 kcal/mol compared with the ciprofloxacin control (−34.42 kcal/mol) (46). If confirmed experimentally, engagement of GyrA could complement the proposed ROS-driven killing environment at the wound surface by limiting bacterial replication and recovery. Second, the phytosterols campesterol and *γ*-sitosterol showed favorable predicted binding to *S. aureus* sortase A (SrtA; Figure 4B), with ΔGbind values of −37.04 and −35.79 kcal/mol, respectively, outperforming the control (−31.77 kcal/mol). Sortase inhibition is a clinically attractive anti-virulence strategy that can reduce surface-protein anchoring and adhesion with a lower risk of resistance selection, which is relevant given the predominance of *Staphylococcus* spp. among our baseline isolates (47, 48).

In the clinical context, such replication arrest would be expected to synergize with the ROS-mediated bactericidal environment already demonstrated for VG111, potentially supporting the rapid infection clearance observed in group 2 (VG111 alone) even without systemic antibiotics (12). Finally, Benzo[h]quinoline (CID610182) and Cinnamyl cinnamate exhibited significantly strong affinities (≈ − 69 kcal/mol) to the LasR regulator (Figure 4C). Cinnamic-acid–related scaffolds have experimentally documented anti–quorum sensing and anti-biofilm effects in *P. aeruginosa,* including studies that combine phenotypic assays with docking-based interaction models, supporting the plausibility that cinnamyl/cinnamate chemistry can engage the quorum-sensing pathway (49, 50). This explanation may help explain the clinical observation of rapid wound healing as observed *in vivo* in VG111-treated groups. In other words, *in silico* results provide a coherent mechanistic hypothesis for the ROS-mediated antimicrobial action and clearance of intact biofilm reported in the previous study (12).

Our study demonstrates several strengths, notably rapid wound contraction, improved BWAT scores, and effective bacterial clearance observed with VG111 compared to PI, as well as the GC–MS analysis revealing a phytochemical profile that may underlie its dual antimicrobial and regenerative mechanisms. However, the study also has limitations. The relatively small sample size limits the generalizability of our findings, and reliance on macroscopic assessments without extensive histopathological or molecular analyses restricts mechanistic resolution. While qualitative swab cultures were used to track infection clearance clinically, quantitative CFU counts were not performed. Future investigations with larger, randomized cohorts and more comprehensive mechanistic studies including ROS quantification at the wound surface, enzyme inhibition assays for predicted targets, and standardized readouts are warranted to further validate and expand upon these promising results.

## Conclusion

The current investigation reveals the evident activity of VG111, a natural product formulation, compared with PI-based wound therapy in 32 canines. The study found that VG111 promoted faster healing than treatment with cefotaxime in combination, suggesting that systemic antibiotics may delay wound healing. GC–MS profiling indicated a chemically diverse formulation enriched in bioactive lipids, lignans, and phytosterols, consistent with a multi-component, multi-mechanism topical effect. Together with prior evidence that VG111 can disrupt intact biofilms and heal chronic wounds, the present findings support VG111 as a promising topical wound dressing candidate.

## Data Availability

The original contributions presented in the study are included in the article/Supplementary material; further inquiries can be directed to the corresponding author.
