# Supplementary material for: A comparative study of wound healing kinetics, efficacy, and profiling of VG111 in canine wounds
Source: Front Vet Sci. 2026 Sep 10;13:1816520. doi: 10.3389/fvets.2026.1816520 (PMC13600891; doi:10.3389/fvets.2026.1816520)
Supplement: Supplementary file 1 [file Supplementary_File_1.docx]

**Supplementary section**

Section 1: Wound healing images of representative cases of each groups

**Supplementary figure 1 (SF1):** Wound healing at different time intervals for case number C18 (group 1: Treated with VG111 + inj cefotaxime)

**SF2:** Wound healing in case number C21 of group 2 on different days (group 2: Treated with VG111 solution only)

**SF3:** Wound healing at different time intervals for case number C36 (group 3: Treated with Povidone-iodine 5% solution + inj cefotaxime)

**SF4:** Wound healing at different time intervals for case number C43 (group 4: Treated with Povidone-iodine 5% solution)
